# Adherence to Therapy, Physical and Mental Quality of Life in Patients with Multiple Sclerosis

**DOI:** 10.3390/jpm11070672

**Published:** 2021-07-16

**Authors:** Alessandra Buja, Guendalina Graffigna, Simona F. Mafrici, Tatjana Baldovin, Carlo Pinato, Umberto Bolzonella, Serena Barello, Alessia Tognetto, Gianfranco Damiani

**Affiliations:** 1 Department of Cardiologic, Vascular and Thoracic Sciences, and Public Health, University of Padova, 35121 Padua, Italy; alessandra.buja@unipd.it (A.B.); tatjana.baldovin@unipd.it (T.B.); umberto.bolzonella@studenti.unipd.it (U.B.); 2 EngageMinds HUB—Consumer, Food & Health Engagement Research Center, Department of Psychology, Università Cattolica del Sacro Cuore, Largo A. Gemelli 1, 20123 Milan, Italy; Guendalina.graffigna@unicatt.it (G.G.); serena.barello@unicatt.it (S.B.); 3 Veneto Institute of Oncology IOV-IRCCS, 35128 Padua, Italy; carlo.pinato@iov.veneto.it; 4 Section of Hygiene, Institute of Public Health, Università Cattolica del Sacro Cuore, Vito 1, 00168 Rome, Italy; alessia.tognetto@gmail.com; 5 Fondazione Policlinico Universitario “A. Gemelli” IRCCS, Largo A. Gemelli 8, 00168 Rome, Italy; gianfranco.damiani@unicatt.it; 6 Department of Life Sciences and Public Health, Università Cattolica del Sacro Cuore, Largo F. Vito 1, 00168 Rome, Italy

**Keywords:** patient adherence, path analysis, socio-economic determinants

## Abstract

Ensuring multiple sclerosis (MS) patients’ adherence to therapy is often challenging, but it is crucial to their survival and health-related quality of life (HRQoL). The aim of the present study was to outline connections between adherence, physical and mental HRQoL, levels of psychological readiness to engage in a treatment, levels of social support, anthropometric, socio-demographic and clinical factors in patients suffering from MS. This cross-sectional study involved a sample of 237 Italian MS patients. A survey was conducted with a structured self-administered online questionnaire using validated measures of quality of life, adherence to therapy and anthropometric, socio-demographic, psychological and clinical variables. A path analysis was used to test the overall structure of the associations between the variables. The pathway indicates a positive association between mental health index and a stronger degree of engagement and being or having been in a long-term relationship. Physical health index was positively associated with age, having an occupation, and having a specific form of MS. Having had relapses in the previous year raised the odds of better adherence to therapy, while an increase in Body Mass Index (BMI) reduced them. Our findings could help in the management of MS patients, promoting behavioral interventions that take the psychological and socio-demographic peculiarities of each patient into account with a view to improving their adherence to therapy.

## 1. Introduction

Among the central nervous system disorders, multiple sclerosis (MS) is the most common cause of permanent disability in young adults, aside from trauma [1], negatively impacting their independence, dignity and future plans [2,3].

Quality of life (QoL) and health-related quality of life (HRQoL) are key parameters in assessing cases of MS [2]. Mitchell [3] reported that QoL is influenced more by psychosocial factors, such as coping, mood tone, autonomy and perceived social support, than by biological variables, such as the extent of lesions evident on magnetic resonance imaging (MRI). In decreasing order of frequency, patients experience anxiety, depression, cognitive decay and anger management problems [4], and higher rates of psychiatric comorbidities have been found among patients with lower incomes [5]. More than half the patients report needs unrelated to their pharmacological therapy that are not identified and managed adequately. These issues generally relate to their personal and professional well-being. Being elderly or single, with a severe degree of progressive disability, and living in rural areas are factors that worsen MS patients’ QoL [6]. 

Ensuring these patients’ adherence to pharmacological therapy is often challenging, but it is crucial to their survival and QoL. According to [7], among patients treated with disease-modifying treatments, almost 75% of patients reported missing at least one dose, and 25% of them had stopped the treatment completely. Poor compliance leads to frequent relapses, increasing the need for hospitalizations and consequently raising healthcare expenditures [8]. Several factors negatively affect patients’ adherence, including cognitive impairment and difficulty administering medication [9], lack of autonomy, shortage of information and poor understanding of the disease, weak expectations of a treatment’s effectiveness, side effects and—more generally—the severity of the disability and comorbidities associated with MS [10]. Psychological distress, also due to the strong sense of uncertainty regarding the course of the disease and the treatment journey, is also considered an important factor influencing patients’ adherence issues [11]. 

Known risk factors for poor adherence have been addressed in a number of studies: MS patients diagnosed with depression are less compliant; people ≥ 45 years old are generally more compliant than younger people; women are less compliant than men [12]; and low levels of formal education and misinformation further diminish patients’ ability and willingness to stick to their prescribed treatment, as well as making it more difficult for them to understand scientific information they are given and to make shared decisions [13]. The social support patients receive is another key factor in MS patients’ adherence to treatment [14,15]. The profiling of MS patients’ psycho-social characteristics in relation to their willingness to adhere to treatment is considered an important frontier for future research [16]. As in other chronic conditions, patients’ psychological readiness to engage in their medical treatment is an important aspect to consider when choosing dedicated counseling and appropriate medical information for these patients [17]. It has been demonstrated that patients’ psychological readiness to engage in their chronic care relies on a complex emotional and cognitive process that can evolve into a typical experiential position of engagement. In this regard, the Patient Health Engagement (PHE) model has proved useful for depicting a patient’s emotional journey along their chronic care pathway and is an important mediator of medical adherence in patients with various chronic conditions [18]. 

Although the importance of shared decision-making and communication between patients and healthcare professionals is unanimously considered important, especially for chronic diseases such as MS that currently have no curative treatments [19], patients with MS report that the feeling that healthcare professionals do not pay attention to their needs is a barrier to the therapeutic process [20].

Further analysis is needed to explore the role of such psycho-social factors in determining MS patients’ adherence to treatment. The aim of the present study was to outline associations between adherence to therapy, physical and mental HRQoL and levels of psychological readiness to engage in a treatment, levels of social support, anthropometric, socio-demographic and clinical factors in patients suffering from MS.

## 2. Materials and Methods

### 2.1. Setting and Participants

The study was conducted on a sample of 237 Italian-speaking MS patients randomly selected from public online groups, Facebook pages, forums and communities dedicated to MS patients (i.e., referring directly to MS in groups’ names or descriptions). The study was advertised online by means of a dedicated post to these groups, describing its objectives and methods, providing assurance that participation in the survey was voluntary and anonymous and that the data would be treated in an aggregate and anonymous form. The post advertising the survey had previously been approved by the administrator of the pages/groups. Patients could volunteer to take part in the study and were under no form of obligation. In order to do so, they were asked to read the information sheet about the study and to sign the consent form. Participants had to meet the following inclusion criteria: Italian speakers suffering from MS over 18 years of age regardless of gender. Refusal to sign the informed consent form or to participate in the study or inability to read written Italian well enough to complete the survey were considered as exclusion criteria.

### 2.2. Survey Development and Main Measures

The survey was based on a structured self-administered online questionnaire (powered by the QUALTRICS online platform, https://www.qualtrics.com/ accessed date: 1 August 2019 to 1 October 2019 ). The questionnaire developed for this study included validated measures and ad hoc items. The forced answering option, which forces the respondent to answer each question in order to proceed through the questionnaire, avoids missing data. A detailed description of the measures included is given below.

- The Patient Health Engagement (PHE-s^®^) Scale [21] consists of 5 ordinal items It has been validated on patients with chronic diseases. We dichotomized the PHE-s^®^ level at its 25th percentile (cut-off = 2): patients with a PHE-s^®^ ≥ 2 expressed a stronger engagement, while patients with a PHE-s^®^ < 2 had a weaker level of engagement.
- The Health-Related Quality of Life (HRQoL) Short-Form 12-item Health Survey [22] assesses generic health outcomes from the patient’s perspective. It has been validated in Italian [23]. The answers to the questions were processed using an algorithm that generates two scores: a Physical Index (Physical Component Summary: PCS), obtained from six of the 12 items, and a Mental Index (Mental Component Summary: MCS) obtained from the other 6. The resulting scores ranged from 1 to 100. We dichotomized both indexes at their 25th percentile, the chosen cut-off being 30.31 for the PCS and 33.25 for the MCS. The higher the scores, the better the patients’ perceived physical and mental health.
- The Modified Social Support Survey (MOS) [24] is a self-report questionnaire devised to measure patients’ perception of their social support. The version used in this study was built to be used specifically with people with multiple sclerosis. On a scale from 0 to 100, a cut-off of 51.3 was chosen, corresponding to the 25th percentile, meaning that patients with a MOS ≥ 51.3 had a more positive perception of their social support.
- The Morisky Green Levine Scale (MGLS) [25] is a validated [26] 4-item self-report measure of patients’ medication-taking behavior. Scores obtained on the MGLS range from 0 to 4. We dichotomized the MGLS level at its 75th percentile (cut-off = 2): patients with a MGLS < 2 had better medication-taking behavior than patients scoring MGLS ≥ 2.
- Health Care Climate Questionnaire (HCCQ–13 item) is a validated self-report questionnaire [27] built to measure the patient’s perception of his doctor’s ability to support him in assuming an autonomous role in the management of the disease. Patients with an HCCQ ≥ 4 have a positive perception of medical support.

### 2.3. Anthropometric, Socio-Demographic and Clinical Characteristics of the Sample

A set of ad hoc items was included in the questionnaire to collect the sample’s anthropometric, socio-demographic and clinical characteristics. These data were also used as screening variables to select a panel of respondents meeting our inclusion criteria. The items assessed the following characteristics: age, gender (male or female), formal education (junior high school or less, high school, university, Masters, PhD), occupational status (employed, unemployed, student) and marital status (single, cohabiting, married, divorced, widowed). The items on respondents’ clinical characteristics concerned the form of MS (relapsing-remitting, RR-MS; secondary-progressive, SP-MS; primary-progressive, PP-MS; or progressive-relapsing, PR-MS) and the occurrence of disease relapses in the previous year.

### 2.4. Statistical Analysis 

The data were analyzed in three steps. In the first, descriptive analyses were conducted, with particular reference to the sample’s anthropometric, socio-demographics, psychological and clinical characteristics. In the second step, a bivariate analysis was run between the anthropometric, socio-demographic, psychological and clinical variables and the MGLS, PCS and MCS scores. All the psychological scales were dichotomized, as explained above. Then, in the third step, a path analysis was used to test the overall structure of the associations between the socio-demographic, psychological and clinical variables. Path analysis via multiple regression was used to test the causal models by examining the relationships between a dependent variable (dichotomized as above) and independent variables. This enabled the simultaneous estimation of several associated regression relationships. A variable could be a dependent variable in one relationship and an independent variable in another. This method enables both the magnitude and the significance of causal connections between variables to be estimated, showing causal mechanisms through which independent variables produce both direct and indirect effects on a dependent variable. The path analysis model was calculated using STATA software, release 13.1.

### 2.5. Ethical Concerns

The study is part of a wider survey named Engagement Monitor, which received the approval of the Ethics Committee of the Department of Psychology at the Università Cattolica del Sacro Cuore. Patients consented to take part in the study and could withdraw whenever they wished. All participants gave their informed consent before being enrolled. The data were collected anonymously and analyzed in aggregate form.

## 3. Results

The anthropometric, socio-demographics, psychological and clinical characteristics of the 237 MS patients are summarized in Table 1. Our sample mainly included women (73%), with an average age of 45 ± 12 years. More than 75% of the participants had attended at least high school. More than 60% had a job. The most frequent type of MS is RR-MS (72%), with no relapses in the last year.

Table 2 shows the association between the patients’ MGLS scores and their anthropometric, socio-demographic, and psychological and clinical characteristics. Statistically significant associations emerged between a greater adherence to therapy and a higher education level (*p* = 0.046), not having offspring (*p* = 0.017), a lower BMI (0.05) and higher MCS (*p* = 0.012) and MOS scores (*p* = 0.036).

Table 3 shows the association between patients’ PCS scores and their anthropometric, socio-demographics, and psychological and clinical characteristics. Statistically significant associations became known between their perceived physical health and a number of socio-demographic and clinical variables. In particular, a higher PCS was associated with younger age (*p* < 0.0001), a higher education (*p* = 0.019), being employed (*p* < 0.0001), not having offspring (*p* = 0.008), a lower BMI (*p* = 0.043), being married (*p* = 0.016) and type of MS (*p* < 0.0001).

Table 4 shows the association between patients’ MCS and their anthropometric, socio-demographics, and psychological and clinical characteristics. Statistically significant associations were identified between perceived mental health and several socio-demographic and psychological variables. In particular, a higher MCS was associated with a higher education (*p* = 0.032), a higher MGLS (*p* = 0.012), a higher PHE-s^®^ (*p* < 0.0001) and higher MOS (*p* = 0.002) scores.

**Figure 1:**
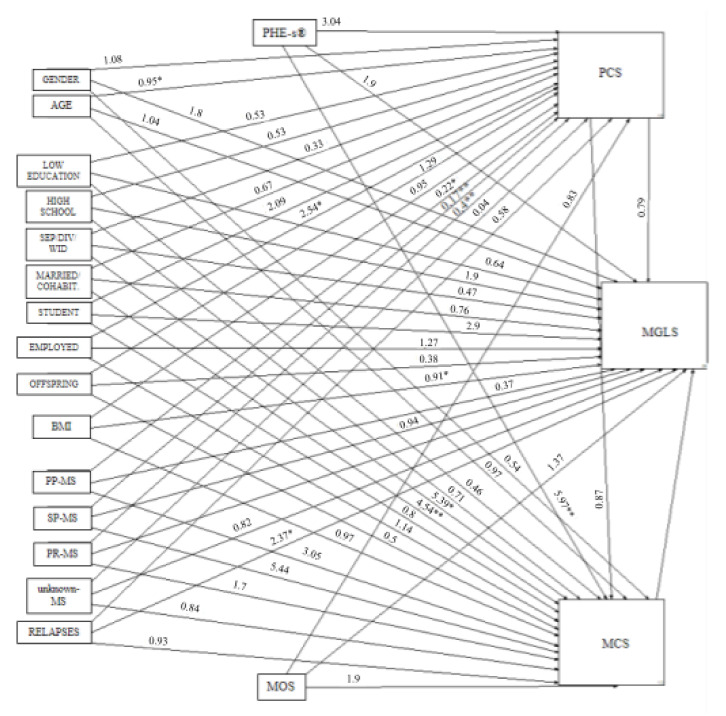
Path analysis. Legend: * *p* < 0.05, ** *p* < 0.01.

Figure 1 shows the results of the path analysis. The pathway indicates that the MGLS was positively associated with BMI and having had relapses in the previous year. There was also a positive association between MCS and PHE-s^®^. Patients who were or had been in a long-term relationship (i.e., cohabiting/married or separated/divorced/widower) seemed to have a higher MCS. PCS was positively associated with age, having an occupation and having a specific form of MS.

The path analysis showed that having had relapses in the previous year raised the odds of a better adherence to therapy (MGLS) (*p* = 0.044), while an increase in BMI reduced them (*p* = 0.017). A stronger degree of engagement (PHE-s^®^) was associated with a better mental health index (MCS) (*p* = 0.002), and being or having been in a long-term relationship (i.e., cohabiting/married or separated/divorced/widower) also enhanced the odds of having a good MCS (*p*= 0.005, 0.048, respectively). Finally, the older the patient’s age the lower the odds of their having a good physical health index (PCS) (*p* = 0.017), while having an occupation was associated with a better PCS (*p* = 0.02). Regarding the form of MS, the primary-progressive, secondary-progressive and progressive-relapsing forms were associated with a lower PCS (*p* = 0.018, 0.003, 0.008, respectively).

## 4. Discussion

The main contribution of our analysis concerns the complex connections it identifies between adherence and physical and mental HRQoL of MS patients with anthropometric, socio-demographic and psychological factors that influence their engagement in their health. In particular, the study revealed an association between adherence to therapy and BMI and relapses in the previous year, between MCS and PHE-s^®^ and between PCS and age, having an occupation and having a specific form of MS.

Our finding that a higher PHE-s^®^ coincides with a better MCS is consistent with previous reports [18]. In fact, PHE-s^®^ could be considered as patients’ ability to elaborate their healthcare experience, to engage actively in their self-management and to have a more positive emotional attitude to their condition. PHE-s^®^ could be conceived as a model of the complex psychological process involved in adjusting to illness, which evolves over time and is influenced by several contextual factors. A high level of health engagement denotes a better psychological adjustment to the disease and the treatment required and a more proactive attitude to self-management. Patients’ level of engagement also relates to their ability to make satisfactory life plans despite the disease, perceiving themselves not just as patients but also as individuals who can promote good health management practices [28]. Profiling patients in terms of their level of engagement, as can be done with the Patient Health Engagement Model, is therefore important for the purposes of counseling and educational initiatives tailored to the different phases of patients’ psychological journey and to nurturing a proactive attitude and adherence to treatment [29]. The present study shows that patients whose disease had relapsed in the year before the survey were more likely to exhibit a better adherence. We interpret this as greater attention to their health condition being prompted by recent painful and stressful episodes. Good compliance is significantly associated with fewer relapses, and fewer hospitalizations and outpatient visits and lower emergency service usage are associated with lower associated costs [30].

Our bivariate and path analyses consistently found that a lower BMI was linked to greater adherence. Compliance can have to do with personal motivation, and a higher BMI may be a sign of a lower self-esteem or depression. A study on obese patients showed that perceived QoL is a mediator between BMI and adherence to therapy [31]. This association needs to be confirmed in further studies but might mean that focused interventions on overweight could lead to better adherence to therapy and clinical prescriptions in MS patients, as suggested by [32] and in other works focusing on chronic diseases such as hypertension [33] and kidney failure [34,35].

Our findings regarding PCS confirm those of previous studies and show the strength of our study design. Our younger respondents reported a better PCS, matching other reports; for instance, Motl [36] found that older MS patients had a worse overall physical functioning and [37] showed an age-related increase in disability. Patients who had an occupation also reported a better PCS. Battaglia [2] also found that patients with a higher PCS tended to be actively working. In our model, three particular forms of MS (PP, SP, and PR) were also associated with a better PCS compared with RR-MS, confirming the report from [38]. 

Some pitfalls could affect the present study. The cross-sectional nature of the data precludes any considerations on potential causal relationships between the variables, so further studies will be needed to test this aspect. Collecting online interviews may also result in a participation bias due to the need to have internet access, an appropriate device and the skills to use them. MS patients are mainly middle-aged, the usual target of these online questionnaires. There may also have been a self-selection bias in the sampling process as the questionnaire was self-administered and the recruitment process relied on patients’ willingness to take part in the study. This may have led to only the psychologically more resilient patients coming forward, making the patient selection not entirely representative of the whole spectrum of MS patients’ psychological experiences.

In conclusion, our findings could help in the management of MS patients, promoting behavioral interventions that take the psychological and socio-demographic peculiarities of each patient into account with a view to improving their adherence. Our results underscore the well-known importance of enhancing patients’ engagement as a strategy to improve their compliance with therapy and help them cope better with such a challenging and unpredictable chronic disease as MS.

## Figures and Tables

**Table 1:** The sample’s anthropometric, socio-demographic, and psychological and clinical characteristics.

Gender (%)	males	26.6
	females	73.4
Age (mean ± SD)		45.3 ± 11.7
Education (%)	junior high school or less	16.5
	high school	52.7
	university/PhD/Masters	30.8
Marital status (%)	single	33.3
	cohabiting/married	59.5
	separated/divorced/widowed	7.2
Occupational status (%)	unemployed	38.4
	employed	54.8
	student	6.8
Offspring (%)	yes	47.7
	no	52.3
Living alone (%)	yes	11
	no	89
BMI (mean ± SD)		24.2 ± 5.5
Type of MS (%)	RR-MS	72.3
	PP-MS	8.2
	SP-MS	8.2
	PR-MS	2.6
	unknown	8.7
Disease relapses in previous year (%)	yes	38.4
	no	61.6
MGLS (%)	higher (≤2)	82.7
	lower (>2)	17.3
PCS (%)	higher (≥30.31)	74.7
	lower (<30.31)	25.3
MCS (%)	higher (≥33.25)	75.1
	lower (<33.25)	24.9
PHE-s^®^ (%)	higher (≥2)	91.1
	lower (<2)	8.9
MOS (%)	higher (≥51.3)	75.1
	lower (<51.3)	24.9

**Table 2:** Bivariate analysis of MGLS with patients’ anthropometric, socio-demographic, and psychological and clinical variables.

MGLS		Higher	Lower	
Dichotomization Cut-Off		≤2	>2	*p*-Value
	*n*		41	
Gender (%)	males	26.5	31.7	0.534
	females	74.5	68.3	
Age (mean ± SD)		45 ± 11.7	46.5 ± 12.3	0.478
Education (%)	junior high school or less	13.8	29.3	0.046
	high school	55.1	41.5	
	university/PhD/Masters	31.1	29.2	
Marital status (%)	single	35.2	24.4	0.212
	cohabiting/married	58.7	63.4	
	separated/divorced/widower	6.1	12.2	
Occupational status (%)	unemployed	35.7	51.2	0.168
	employed	56.6	46.3	
	student	7.7	2.4	
Offspring (%)	yes	43.8	65.8	0.017
Living alone (%)	yes	10.7	12.2	0.785
BMI (mean ± SD)		23.8 ± 5.2	26 ± 6.5	0.05
Type of MS (%)	RR -SM	71.4	65.9	0.503
	PP-SM	7.7	12.2	
	SP-SM	9.2	7.3	
	PR-SM	3.1	0	
	unknown		14.6	
Disease relapses in previous year (%)	yes	40.8	26.8	0.134
PCS (%)	higher (≥30.31)	75	73.1	0.962
	lower (<30.31)	25	26.8	
MCS (%)	higher (≥33.25)	78.6	58.5	0.012
	lower (<33.25)	21.4	41.5	
PHE-s^®^ (%)	higher (≥2)	92.9	82.9	0.083
	lower (<2)	7.1	17	
MOS (%)	higher (≥51.3)	78.1	61	0.036
	lower (<51.3)	21.9	39	

**Table 3:** Bivariate analysis of PCS with patients’ anthropometric, socio-demographic, and psychological and clinical variables.

PCS		Higher	Lower	
Dichotomization Cut-Off		≥30.31	<30.31	*p*-Value
	*n*	177	60	
Gender (%)	males	24.3	33.3	0.229
	females	75.7	66.7	
Age (mean ± SD)		42.7 ± 10.3	53.1 ± 12.5	<0.0001
Education (%)	junior high school or less	13.6	25	0.019
	high school	51.4	56.7	
	university/PhD/Masters	35	18.3	
Marital status (%)	single	38.4	18.3	0.016
	cohabiting/married	55.4	71.7	
	separated/divorced/widowed	6.2	10	
Occupational status (%)	unemployed	29.9	63.3	<0.0001
	employed	61.6	35	
	student	8.5	1.7	
Offspring (%)	yes	42.4	63.3	0.008
Living alone (%)	yes	11.3	10	0.969
BMI (mean ± SD)		23.7 ± 5	25.6 ± 6.6	0.043
Type of MS (%)	RR-SM	80.2	41.7	<0.0001
	PP-SM	5.1	18.3	
	SP-SM	4.5	21.7	
	PR-SM	0.6	8.3	
	unknown	9.6	10	
Disease relapses in previous year (%)	yes	36.7	43.3	0.449
MGLS (%)	higher (≤2)	83	81.7	0.962
	lower (>2)	16.9	18.3	
MCS (%)	higher (≥33.25)	75.1	75	1
	lower (<33.25)	24.9	25	
PHE-s^®^ (%)	higher (≥2)	93.2	85	0.09
	lower (<2)	6.8	15	
MOS (%)	higher (≥51.3)	75.7	73.3	0.846
	lower (<51.3)	24.3.	26.7	

**Table 4:** Bivariate analysis of MCS with patients’ anthropometric, socio-demographic, and psychological and clinical variables.

	MCS	Higher	Lower	
Dichotomization Cut-Off		≥33.25	<33.25	*p*-Value
	*n*	178	59	
Gender (%)	males	29.8	16.9	0.078
	females	70.2	83.1	
Age (mean ± SD)		45.3 ± 11.8	45.4 ± 11.6	0.948
Education (%)	junior high school or less	33.1	23.7	0.032
	high school	12.9	27.1	
	university/PhD/Master	53.9	49.2	
Marital status (%)	single	29.2	45.8	0.059
	cohabiting/married	63.5	47.5	
	separated/divorced/widowed	7.3	6.8	
Occupational status (%)	unemployed	36.5	44.1	0.586
	employed	56.7	49.2	
	student	6.7	6.8	
Offspring (%)	yes	53.4	49.2	0.68
Living alone (%)	yes	91.6	81.4	0.053
BMI (mean ± SD)		24.1 ± 5.3	24.4 ± 6.1	0.741
Type of MS (%)	RR-SM	69.1	74.6	0.164
	PP-SM	9.6	5.1	
	SP-SM	10.7	3.4	
	PR-SM	2.8	1.7	
	unknown	7.9	15	
Disease relapses in previous year (%)	yes	62.9	57.6	0.568
MGLS (%)	higher (≤ 2)	86.5	71.2	0.012
	lower (>2)	13.5	28.8	
PCS (%)	higher (≥33.25)	74.7	74.6	1
	lower (<33.25)	25.3	25.4	
PHE-s^®^ (%)	higher (≥2)	96.6	74.6	<0.0001
	lower (<2)	3.4	25.4	
MOS (%)	higher (≥51.3)	80.3	59.3	0.002
	lower (<51.3)	19.7	40.4	
